# Association of *IL‐10*‐1082A/G polymorphism with cardiovascular disease risk: Evidence from a case–control study to an updated meta‐analysis

**DOI:** 10.1002/mgg3.888

**Published:** 2019-09-30

**Authors:** Shijuan Lu, Jianghua Zhong, Kang Huang, Honghao Zhou

**Affiliations:** ^1^ Department of Clinical Pharmacology Xiangya Hospital, Central South University and Institute of Clinical Pharmacology, Central South University, Hunan Key Laboratory of Pharmacogenetics Changsha P.R. China; ^2^ Department of Cardiology Haikou People’s Hospital, Central South University Xiangya School of Medicine Affiliated Haikou Hospital Haikou P.R. China

**Keywords:** an updated meta‐analysis, cardiovascular disease, interleukin‐10, polymorphism

## Abstract

**Background:**

Previous studies have generated controversial results about the association of interleukin 10 (*IL‐10*) gene polymorphisms (−1082G/A) in the progression of cardiovascular disease (CVD). Therefore, this study processed a systemic meta‐analysis to verify this association.

**Methods:**

The publication studies on the *IL‐10* (−1082G/A) polymorphism and CVDs risk were obtained by searching PubMed and Embase databases. We analyzed the genotype data for meta‐analysis. The results were evaluated by odds ratios (ORs) and 95% confidence intervals (CIs). Meanwhile, our meta‐analysis was also performed sensitivity analyses, heterogeneity test, and identification of publication bias.

**Results:**

The present meta‐analysis suggested that the risk with allele G is lower than with allele A for CVD. The G allele of *IL‐10* (−1082) could increase the risk of CVDs in the 31 case–control studies for all genetic models. (OR = 1.10, 95% CI: 1.04–1.15 for the allele model A vs. G; OR = 0.87, 95% CI: 0.72–1.04 for the dominant model GG+AG vs. AA; OR = 1.03, 95% CI: 1.02–1.05 for the recessive model GG vs. AG + AA; OR = 1.06, 95% CI = 1.03–1.10 for the homozygote comparison model GG vs. AA; and OR = 0.88, 95% CI = 0.73–1.06 for the heterozygote comparison model AG vs. AA).

**Conclusions:**

In genetic models, the association between the *IL‐10* (−1082G/A) polymorphism and CVDs risk was significant. This meta‐analysis proposes that the *IL‐10* (−1082G/A) polymorphism may serve as a risk factor for CVDs.

## 1 INTRODUCTION

Cardiovascular diseases (CVDs) as the type of age‐related disease including coronary artery disease (CAD), stroke, hypertension, and cerebral infarction, which are one of the main causes of morbidity and mortality in the world (Chen et al., 2017; Mozaffarian et al., 2016; Song et al., 2017; Sun et al., 2017; Yu et al., 2017; Yusuf, Reddy, Ôunpuu, & Anand, 2001; Zhao et al., 2016). The clinical risk factors about CVDs have been established for many years, including obesity, hypertension, diabetes, and an inactive lifestyle (Cooper et al., 2000; Kaess et al., 2015; Yusuf et al., 2001). However, the pathological statistical concept suggested that the complex molecular basis of CDV was related to a wide range of biological pathways, involving lipid and glucose metabolism, vascular repair and angiogenesis (Gaziano et al., 2006). Several studies have shown that the etiology and pathogenesis of CVDs were likely to comprise a multifactorial disorder resulting from environmental and genetic factors (Cooper, 1999). Apart this, more and more researches identified that inflammatory molecules might play a central role in the pathogenesis of Cardiovascular diseases as well (Marousi, Ellul, et al., 2011).

The occurrence and development of arterial thrombotic diseases are involved in the inflammation (Meuwissen et al., 2004). Inflammatory cytokines are recognized as dysregulated in aging and age‐related disease (Liu, Wang, & Jiang, 2017). Interleukin 10 (*IL‐10*, OMIM: 226,990), as a potent immunoregulatory cytokine, is a newly discovered cytokine in recent years, which is widely known for its anti‐inflammatory and B‐cell stimulating function (Franceschi & Campisi, 2014; Rea et al., 2018). According to the chromosomal location and functional relevance, *IL‐10* is a multifunctional cytokine that could not only inhibit the synthesis of proinflammatory cytokines but also downregulate antigen presentation and macrophage activation (Lee, Kim, & Song, 2014). Scientific literature reported that *IL‐10* was related to the pathogenesis and development of coronary artery inflammation (Heeschen et al., 2003a), and have been considered a candidate gene for kind of cardiovascular diseases (CVD) (Liu, Hui‐Min, Yang, & Geriatrics, 2017), including vasculitis, cerebral infarction, atrial fibrillation, atherosclerotic, and stroke, but the results are still controversial. Therefore, this meta‐analysis was performed to obtain a more precise conclusion and to make a better understanding of *IL‐10* (−1082 G/A) single‐nucleotide polymorphism (SNP) with CVDs risk.

Currently, many epidemiological studies have focused on the relationship between *IL‐10* (−1082 G/A) SNP and CVDs risk, including CAD, stroke, hypertension, and cerebral infarction, but the results are still controversial (Afzal et al., 2012; Ben‐Hadj‐Khalifa et al., 2010; Karaca, Kayıkçıoğlu, Onay, Gündüz, & Ozkınay, 2011b; Marousi, Ellul, et al., 2011; Zhang, Pan, Ran, & Bing‐Xun, 2007). Furthermore, a single study might be too underpowered to provide accurate conclusion because of relatively small sample size. In order to reach a reliable conclusion, we designed this meta‐analysis to assess the relationship of *IL‐10*(−1082 G/A) SNP with the risk of CVDs.

## 2 MATERIALS AND METHODS

### 2.1 Inclusion and exclusion criteria

We executed an extensive search for databases including PubMed, Embase, and Web of Science to confirm relevant research to analyze the association between the *IL‐10* polymorphisms and CVDs risk. The last report was updated on April 17, 2017. When included in the analysis, qualified researches must meet the following criteria: (1) the association studies about the *IL‐10* polymorphism and susceptibility to related CVDs must follow case–control study strategies; (2) all patients meet the diagnostic criteria for CVDs in the candidate studies; (3) There is enough available data to calculate the odds ratio (OR) and 95% confidence interval (CI). The major exclusion criteria for studies were: (a) not a case–control study; (b) studies that have been republished; and (c) no feasibility data studies. This meta‐analysis was performed conforming to the Preferred Reporting Items for Systematic Reviews and Meta‐analyses (PRISMA) guidelines. Finally, data for meta‐analysis were available from 31 studies, including 10,502 cases and 7,865 controls 61 (Afzal et al., 2012; Benhadjkhalifa et al., 2010; Cruz et al., 2013; Donger et al., 2001; Elsaid, Abdelaziz, Elmougy, & Elwaseef, 2014; Fragoso et al., 2011; He et al., 2015; Ianni et al., 2012; Jiang, Lin, Zhang, Chen, & Liu, 2015; Jin, Peihua, Jiemin, Yi, & Hailiang, 2011; Karaca, Kayıkçıoğlu, Onay, Gündüz, & Ozkınay, 2011a; Koch et al., 2001; Kumar et al., 2016; Li, Gao, He, & Zhang, 2016; Lin, 2009; Lio et al., 2004; Liu, Li, Zhu, & He, 2017; Lorenzová et al., 2007; Marousi, Ellul, et al., 2011; Munshi et al., 2010; O'Halloran, Stanton, O'Brien, & Shields, 2006; Ozkan, Sılan, Uludağ, Degirmenci, & Karaman, 2015; Seifart et al., 2005; Babu et al., 2012; Sultana et al., 2011; Tabrez et al., 2017; Tuttolomondo et al., 2012a; Yu et al., 2012; Zhang et al., 2007; Zheng et al., 2014).

### 2.2 Data extraction

Primary investigators used standardization requirements to extract qualified research from the database. They organized the data into tables and submitted them to coauthors for review to ensure that data are strict and reasonable. We collected the following information from the retrieved literature: first author's name, publication year, control source, country and ethnicity of population, the methods of genotype, the number of cases and controls, the distribution of genotype in cases and controls, and the *p* value for HWE (Hardy–Weinberg Equilibrium) in controls. Different ethnicity was categorized as Asians, Caucasians, and Mixed.

### 2.3 Statistical analyses

The *IL‐10* (−1082 G/A) polymorphisms and CVDs risk was assessed by OR and the corresponding 95% CI for each study. Different ORs (95% CI) were calculated using the following models: the allele model (G vs. A), the dominant model (GG + AG vs. AA), the recessive model (GG vs. AG + AA), the homozygote model (GG vs. AA), and the heterozygote model (GA vs. AA). Using the Cochran’s *Q* statistic and *I* ^2^ test to evaluated the statistical heterogeneity between eligible studies, which was considered as significant when *p*_Q_＜0.1 or *I* ^2^ > 50%. A fixed‐effect model (the Mantel–Haenszel method) was adopted if *p* > .1 and *I* ^2^ < 50%, and a random‐effect model (DerSimonian–Laird method) was used if *p* < .1 and *I* ^2^ > 50%. Furthermore, we conducted subgroup analysis based on ethnicity (Asian, Caucasian, or Mixed) and disease subtype (CAD, stroke, and cerebral infarction) to explore the sources of heterogeneity. All statistical analyses were performed using software STATA 15.0 (StataCorp LP).

## 3 RESULTS

### 3.1 Eligible studies

**Figure 1:**
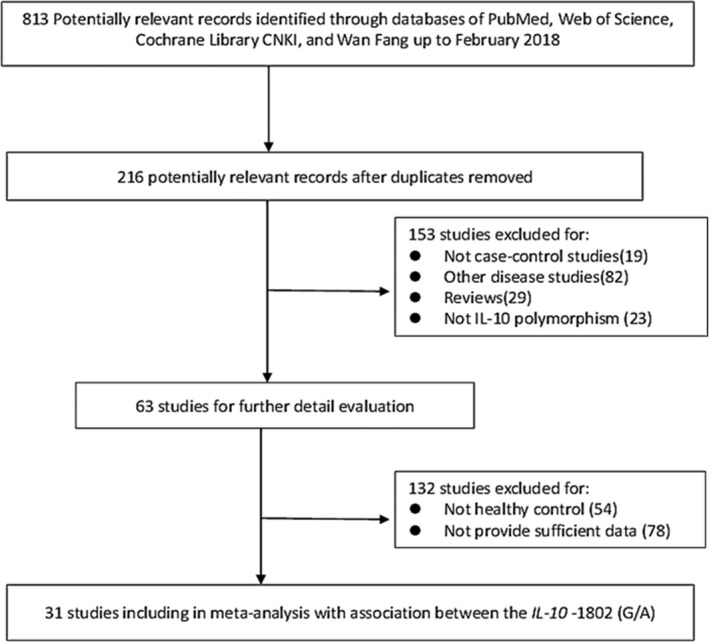
Flow chart showing study selection procedure

As shown in Figure 1, the process of literature retrieval and exclusion is listed. In general, the preliminary comprehensive search was identified 813 relevant articles. According to the inclusion and exclusion criteria, this meta‐analysis is involving 31 case–control studies with 18,367 total sample sizes (10,502 cases and 7,865 controls).

The characteristics of the studies are listed in Table 1. *IL‐10* (−1082 G/A) genotype distributions in the controls from all studies has carried on HWE (Table 2). The 31 studies identified in this meta‐analysis including 14 studies of Caucasians, 15 studies of Asians, and two studies from mixed population. Among these researches, patients were mostly recruited in referral centers with CAD, stroke, cerebral infarction and myocardial infarction, and the controls have not any obvious disease.

**Table 1:** Characteristics of eligible studies included in the meta‐analysis

First author (year)	Disease	Control source	Country	Ethnicity	Matching	Genotyping
						method
Koch et al. (2001)	Coronary artery disease and myocardial infarction	HB	Germany	White	Age, sex	AS‐PCR
Donger et al. (2001)	Myocardial infarction	NA	Mixed	White	Age, sex	PCR‐SSCP
Lio et al. (2004)	Cardiovascular diseases	HB	North Italy	White	Age	PCR‐SSP
Lio et al. (2004)	Coronary heart disease	HB	South Italy	White	Age	PCR‐SSP
Seifart et al. (2005)	Cardiovascular diseases	PB	Germany	White	NA	PCR‐RFLP
O’Halloran et al. (2009)	Coronary artery disease	NA	Ireland	White	NA	AS‐PCR
Zhang et al. (2007)	Cerebral infarction	PB	China	Asian	NA	PCR‐RFLP
Lorenzová et al. (2007)	Myocardial infarction	PB	Czech Republic	White	Age	PCR‐RFLP
Lin et al. (2009)	Cerebral infarction	HB	China	Asian	Age, sex	ARMS‐PCR
Ben‐Hadj‐Khalifa et al. (2010)	Coronary artery disease	NA	Tunisia	White	Age, sex	AS‐PCR
Munshi et al. (2010)	Ischemic stroke,	PB	India	Asian	Age, sex	ARMS‐PCR
Karaca et al. (2011b)	Coronary artery disease	NA	Turkey	White	Age	PCR‐RFLP
Marousi, Ellul, et al. (2011)	Ischemic stroke	PB	Greece	White	Age, sex	RT‐PCR
Sultana et al. (2011)	Cerebral infarction	PB	India	Asian	Age	ARMS PCR
Jin et al. (2011)	Cerebral infarction	HB	China	Asian	Age	RFLP‐PCR
Fragoso et al. (2011)	Acute coronary syndrome	HB	Mexico	Mixed	Age, sex	RT‐PCR
Babu et al. (2012)	Cardiovascular diseases	NA	India	Asian	Age, sex	ARMS‐PCR
Afzal et al. (2012)	Coronary artery disease	HB	Pakistan	Asian	Age	ARMS‐PCR
Ianni et al. (2012)	Myocardial infarction	NA	South Italy	White	NA	TaqMan
Tuttolomondo et al. (2012a)	Ischemic stroke	HB	Italy	White	Age	ASO‐PCR
Yu et al. (2012)	Ischemic heart disease	PB	Korea	Asian	NA	Pyrosequencing
Cruz et al. (2013)	Myocardial ischemia	NA	Mexico	Mixed	NA	TaqMan
Elsaid et al. (2014)	Cardiovascular	NA	Egypt	White	NA	PCR
Zheng et al. (2014)	Atrial fibrillation	PB	China	Asian	NA	PCR‐RFLP
He et al. (2015)	Ischemic stroke	PB	China	Asian	Age	PCR‐RFLP
Jiang et al. (2015)	Ischemic stroke	HB	China	Asian	Age, sex	PCR‐RFLP
Ozkan et al. (2015)	Ischemic stroke	HB	Italy	White	Age	RT‐PCR
Kumar et al. (2016)	Ischemic stroke	PB	India	Asian	Age	PCR‐RFLP
Li et al. (2016)	Ischemic heart disease	PB	China	Asian	Age, sex	PCR‐RFLP
Liu, Hui‐Min, et al. (2017)	Ischemic heart disease	PB	China	Asian	Age, sex	PCR‐LDR
Tabrez et al. (2017)	Cardiovascular diseases	HC	KAUH	Asian	NA	PCR

**Table 2:** Genotype distribution among studies included in the meta‐analysis

First author (year)	Sample size	Case			Control			HWE
	(Case/Control)	AA	AG	GG	AA	AG	GG	
Koch et al. (2001)	1,791/340	540	874	377	105	161	74	0.407
Donger et al. (2001)	1,107/1,082	242	486	256	231	477	244	0.944
Lio et al. (2004)	142/153	60	52	30	30	75	48	0.942
Lio et al. (2004)	90/110	44	29	17	28	56	26	0.846
Seifart et al. (2005)	104/243	19	59	25	86	115	42	0.739
O'Halloran et al. (2006)	1,598/386	324	784	490	77	138	117	0.004
Zhang et al. (2007)	204/131	202	2	0	120	14	0	0.523
Lorenzová et al. (2007)	284/568	90	98	40	207	255	106	0.083
Lin (2009)	181/90	153	28	0	83	32	0	0.083
Ben‐Hadj‐Khalifa et al. (2010)	291/291	76	108	101	52	100	76	0.088
Munshi et al. (2010)	480/470	92	241	147	63	218	189	0.991
Karaca et al. (2011b)	86/88	20	44	22	21	44	23	0.996
Marousi, Antonacopoulou, et al. (2011)	145/145	47	71	27	53	71	21	0.723
Sultana et al. (2011)	238/226	154	44	40	163	47	16	0.000
Jin et al. (2011)	189/92	161	27	1	78	12	2	0.087
Fragoso et al. (2011)	389/302	211	142	36	164	113	25	0.38
Babu et al. (2012)	651/432	318	260	73	170	188	74	0.079
Afzal et al. (2012)	93/99	6	77	10	4	92	3	0.000
Ianni et al. (2012)	267/321	68	141	56	78	88	73	0.000
Tuttolomondo et al. (2012a)	96/48	58	14	24	20	17	11	0.065
Yu et al. (2012)	173/313	150	22	1	275	38	0	0.253
Cruz et al. (2013)	149/248	55	83	11	125	106	17	0.387
Elsaid et al. (2014)	108/143	2	49	22	8	85	5	0.000
Zheng et al. (2014)	117/100	84	27	6	55	35	10	0.221
He et al. (2015)	260/260	41	124	95	29	108	123	0.475
Jiang et al. (2015)	181/115	153	28	0	83	32	0	0.083
Ozkan et al. (2015)	42/48	11	26	5	19	18	11	0.113
Kumar et al. (2016)	250/250	11	77	162	4	37	209	0.127
Li et al. (2016)	335/335	54	151	130	34	143	158	0.844
Liu, Hui‐Min, et al. (2017)	386/386	313	68	5	308	75	3	0.498
Karami, Zabihzadeh, Shams, and Saki Malehi (1002)	75/50	1	66	8	40	1	9	0.000

### 3.2 Meta‐analysis databases

**Figure 2:**
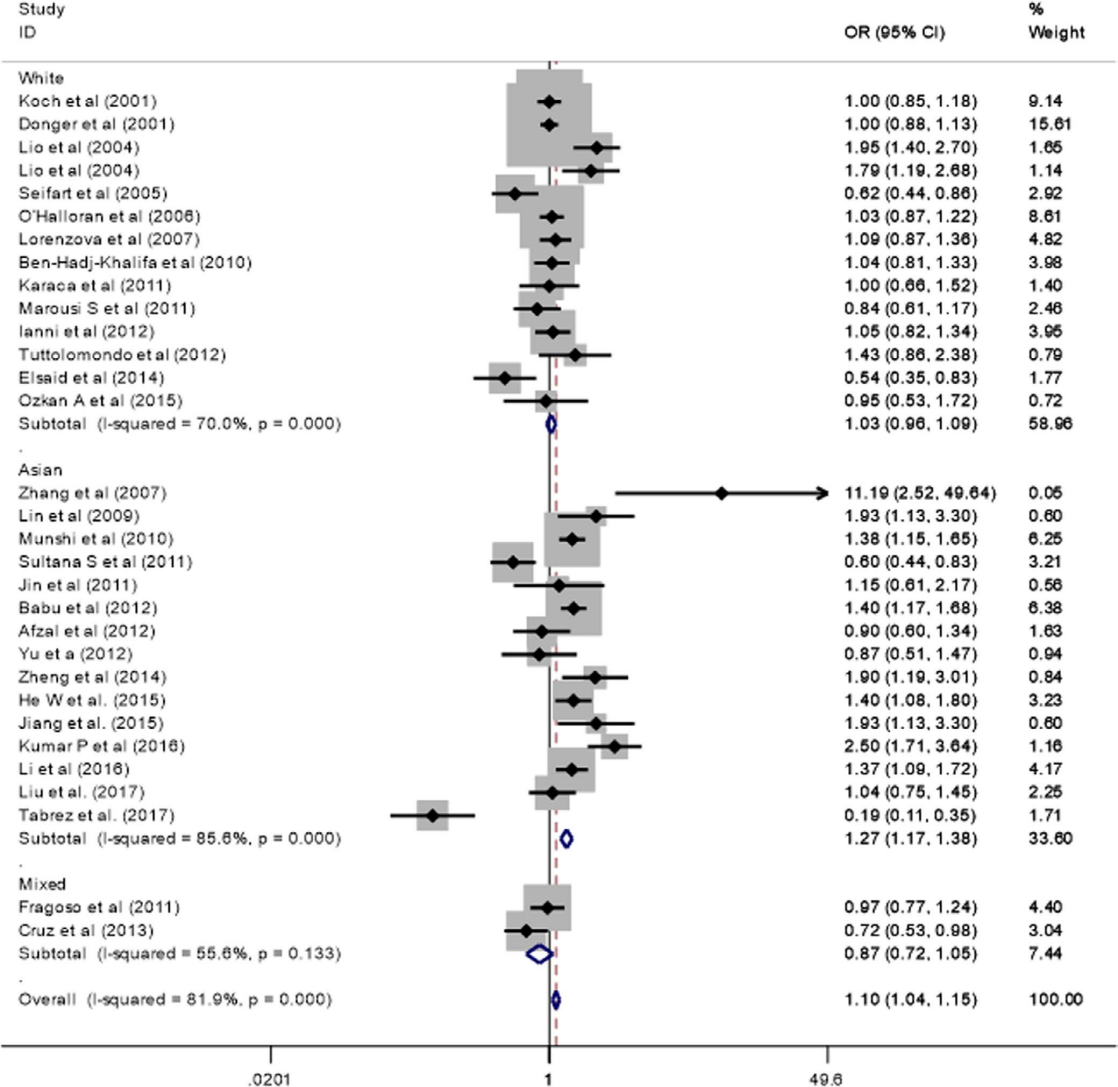
Forest plots for the *IL‐10*‐1082A/G polymorphism and cardiovascular disease in the allele model

As shown in Tables 3 and 4, the results including the association of the *IL‐10* (−1082 G/A) polymorphism and CVDs risk, as well as homozygote test and heterogeneity test. The combined results showed that the variant genotypes were related to increase the CVDs risk in different genetic models (OR = 1.06, 95% CI = 1.03–1.10 for the homozygote comparison model GG vs. AA; OR = 0.88, 95% CI = 0.73–1.06 for the heterozygote comparison model AG vs. AA; OR = 1.10, 95% CI: 1.04–1.15 for the allele model A vs. G; OR = 0.87, 95% CI: 0.72–1.04 for the dominant model GG + AG vs. AA; and OR = 1.03, 95% CI: 1.02–1.05 for the recessive model GG vs. AG + AA). The forest plots for −1082 G/A in the allele model are shown in Figure 2. In subgroup analyses by ethnicity, our results were similar to the Caucasian population.

**Table 3:** Stratified analyses of the association between IL‐10‐1082A/G polymorphisms and cardiovascular disease risk

Variables	Allele model			Dominant model			Recessive model		
	G/A			(GG/AG vs. AA)			(GG vs. AG/AA)		
	OR (95% CI)	*p*_OR_	*p*_Het_	OR (95% CI)	*p*_OR_	*p*_Het_	OR (95% CI)	*p*_OR_	*p*_Het_
Total	1.10 (1.04–1.15)	0.000	0.000	0.87 (0.72–1.04)	0.036	0.000	1.03 (1.02–1.05)	0.000	0.000
Ethnicity									
Asian	1.27 (1.17–1.38)	0.000	0.000	0.73 (0.54–0.99)	0.000	0.000	1.31 (0.95–1.81)	0.000	0.000
Caucasian	1.03 (0.96–1.09)	0.446	0.000	0.95 (0.76–1.20)	0.754	0.000	1.03 (0.85–1.25)	0.629	0.930
Mix	0.87 (0.72–1.05)	0.154	0.133	1.29 (0.76–2.22)	0.118	0.036	0.90 (0.58–1.39)	0.000	0.000
Source of control									
HB	1.10 (0.99–1.22)	0.063	0.000	0.88 (0.76–1.02)	0.080	0.000	1.12 (0.92–1.36)	0.246	0.150
PB	1.18 (1.08–1.28)	0.000	0.000	0.87 (0.76–0.99)	0.032	0.000	1.30 (1.14–1.49)	0.000	0.000

Note

*p*_OR_: *p* value from the odd ratio and obtained from *Z* test; *p*_Het_: *p* value from the heterogeneity and obtained from the chi‐square test.

**Table 4:** Heterogeneity and homogeneity analyses of the association between *IL‐10*‐1082A/G polymorphisms and cardiovascular disease risk

Variables	Homozygote (GG vs. AA)			Heterogeneity (AG vs. AA)		
	OR (95% CI)	*p*_OR_	*p*_Het_	OR (95% CI)	*p*_OR_	*p*_Het_
Total	1.06 (1.03–1.10)	0.009	0.000	0.88 (0.73–1.06)	0.205	0.000
Ethnicity						
Asian	0.81 (0.50–1.31)	0.000	0.000	0.75 (0.56–1.00)	0.003	0.000
Caucasian	0.95 (0.74–1.21)	0.464	0.00	0.97 (0.74–1.26)	0.853	0.000
Mix	1.22 (0.77–1.92)	0.406	0.588	1.29 (0.72–2.33)	0.138	0.028
Source of control						
HB	0.84 (0.68–1.05)	0.14	0.000	0.90 (0.77–1.04)	0.165	0.000
PB	0.82 (0.69–0.99)	0.039	0.000	0.87 (0.75–0.99)	0.047	0.005

Note

*p*_OR_: *p* value from the odd ratio and obtained from *Z* test; *p*_Het_: *p* value from the heterogeneity and obtained from the chi‐square test.

### 3.3 Test of heterogeneity

Using the chi‐square test and fixed effects model, we observed the statistically significant heterogeneity in trials (Allele model G vs. A: *p* = .000, *I* ^2^ > 50%; dominant model GG/AG vs. AA: *p* = .000, *I* ^2^ ＞ 50%; recessive model GG vs. AG/ AA: *p* = .000, *I* ^2^ > 50%; homozygote comparison model GG vs. AA: *p* = .000, *I* ^2^ > 50%; heterozygote comparison model GA vs. AA: *p* = 0.000, *I* ^2^ > 50%) (Tables 3 and 4). Thus, the wider CIs will be generated by the random‐effect model.

### 3.4 Sensitivity analysis and Bias diagnostics

**Figure 3:**
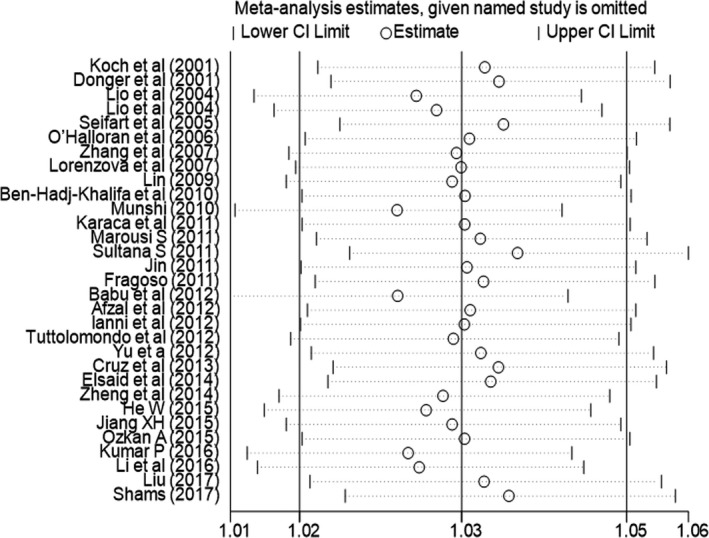
Sensitivity analyses of the summary odds ratio coefficients for the allele model in the overall meta‐analysis

**Figure 4:**
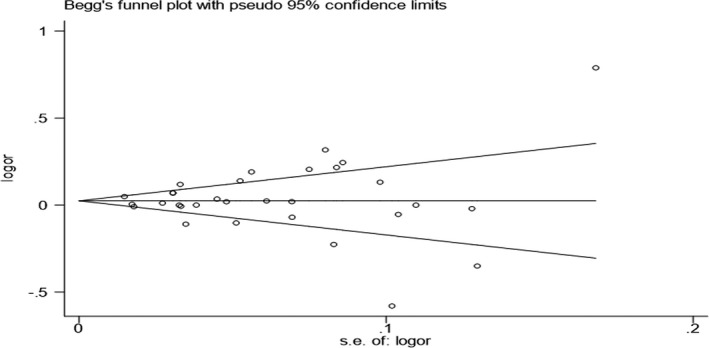
Begg's funnel plot from the meta‐analysis of cardiovascular disease risk and *IL‐10*‐1082A/G polymorphism in the allele model

To evaluate the stability of the pooled results, sensitivity analysis was performed. The result showed that there were no substantial changes in ORs after canceling each study (Figure 3). As to the publication bias, it was estimated by the Begg's funnel plots and Egger's tests. The funnel plot shapes did not reveal obvious evidence of asymmetry (Figure 4). Additionally, according to the result of Egger's tests, the *p* values greater than 0.05 (G vs. A: *p* = .814; GG/AG vs. AA: *p* = .789; GG vs. AG/ AA: *p* = .253; GG vs. AA: *p* = .312; GA vs. AA: *p* = .855), which providing statistical evidence to the funnel plots’ symmetry.

## 4 DISCUSSION

Cardiovascular disease is broadly testified as atherosclerosis, underlying vascular aberrations, as well as formation of coronary plaques (Malik et al., 2004; Strazzullo, D’Elia, Kandala, & Cappuccio, 2009; Vasan, 2006). Many studies identified that the potential influence of inflammatory cascade in many kinds of CVD. Inflammatory responses could trigger and accelerate vascular injury and CVDs risk (Lakka et al., 2002; Malik et al., 2004). Therefore, the pathogenesis of CVDs have been deemed as both genetic and inflammatory pathways, which modulated by various inflammatory cytokines. Genetic factors are regarded as strong determinants of CVDs (Brigitta, 2007; Mckusick, 1959). According to the chromosomal location and functional relevance, *IL‐10* is a multifunctional cytokine that has been considered as a candidate gene for kinds of CVD (Couper, Blount, & Riley, 2008; Dopheide et al., 2015; Eskdale, Wordsworth, Bowman, Field, & Gallagher, 2010; Heeschen et al., 2003a). Therefore, this meta‐analysis was performed to obtain a more precise conclusion and to make a better understanding of *IL‐10* (−1082 G/A) SNP with CVDs risk.

For this meta‐analysis, we aimed to identify the dispute about the role of *IL‐10* (−1082 G/A) in CVD risk. The results showed that there exists a significant relationship between *IL‐10* (−1082 G/A) and CVDs risk. This study was conducted by critically evaluating 31 individual case–control studies involving the *IL‐10* (−1082 G/A) polymorphism and CVDs risk. We found that the *IL‐10* (−1082 G/A) was associated with an increased risk of CVDs among Asians. As far as we know, this updated meta‐analysis includes the largest samples and the most cogent conclusions.

In our study, we observed that the allele and genotype frequencies of *IL‐10* (−1082A/G) were associated with CVDs risk, which was consistent with a present study, as reported by Yang et al. (Xuan, Wang, Zhi, Li, & Wei, 2016). One meta‐analysis study observed that the allele and genotype frequencies of *IL‐10* (−1082A/G) were not associated with ischemic stroke risk (Liu, Hui‐Min, et al., 2017). However, the powerful relationship between *IL‐10* (−1082A/G) and ischemic stroke has been found in the Italian population (Tuttolomondo et al., 2012a). Li et al. reported that *IL‐10* −1082G/A was related to increase the atherosclerotic risk (Chao, Lei, & Fei, 2014). Wang et al. observed that *IL‐10* (−1082A/G) polymorphism was significantly associated with increasing the cerebral infarction risk in Asians (Fan et al., 2016). Based on the Asian population, several studies have indicated the positive or null relationship of *IL‐10* (−1082A/G) with CVDs, and the results remain controversial. Although previous individual studies have reported an association, the overall result of the present analysis support the relationship between *IL‐10* −1082G/A polymorphism and CVD risk in all genetic models. There are three potential reasons can explain the difference among these findings. First, the relatively small sample sizes of included studies might lead to false positive or negative results. Second, the inconsistent results could result from different genotype frequencies among enrolled subjects, especially in different ethnic groups. Third, due to the complicated pathogenesis of CVDs, it is difficult to explain that the SNP in a single gene could increase risk of CVD without a contribution of other polymorphic susceptibility genes.

Limitations also exist in our study. First, the study lack of detailed information in patients. Second, the studies with small sample size (<100 cases and controls) may overestimate the relationship. Third, the origins of heterogeneity may contain many factors, such as the diverse characteristics of the control group and the different methods of diverse genotyping. Finally, the human *IL‐10* production is regulated by the complicated interaction between gene and environment, the effect of single genetic mutation is limited. So the evidence offered by this meta‐analysis should be accepted with caution. However, our study also exist some advantages: the reasonable‐designed search and selection method could increase the statistical power and the accuracy of the results, at the same time, the results did not show any evidence of publication bias.

In conclusion, the results of our study indicate that the *IL‐10* −1082A/G polymorphism is associated with an increased risk of CVDs. In subsequent studies, we will conduct functional studies to confirm our conclusions.

## CONFLICT OF INTEREST

The authors have no conflict of interest to report.
